# Traumatic Brain Injury: Advances in Diagnostic Techniques and Treatment Modalities

**DOI:** 10.3390/jcm14207145

**Published:** 2025-10-10

**Authors:** Lori Zarmer, Maaz S. Khan, Glenn Islat, Hanan Alameddin, Maria Massey, Saki Kazui, Rabail Chaudhry

**Affiliations:** 1 Department of Anesthesiology and Pain Medicine, Banner University Medical Center, University of Arizona COM-T, Tucson, AZ 85724, USA; lorizarmer@arizona.edu (L.Z.);; 2 Department of Anesthesiology, Critical Care and Pain Management, Hospital for Special Surgery and Department of Anesthesiology, Weill Cornell Medical College, New York, NY 10021, USA; maazkhan03441@gmail.com

**Keywords:** traumatic brain injury, TBI, trauma, brain injury, head injury, concussion, critical care

## Abstract

**Background/Objectives:** Traumatic brain injury (TBI) is a major global cause of death and disability, with long-term cognitive, behavioral, and functional consequences. Despite its high burden, management is complicated by heterogeneous presentations and limited evidence. This review summarizes recent advances in monitoring, therapeutic strategies, neuroprotection, and rehabilitation, while highlighting future directions toward individualized and globalized care. **Methods**: This paper is a narrative review of clinical trials, systematic reviews, and observational studies, focusing on invasive and non-invasive monitoring, pharmacologic and non-pharmacologic interventions, neuroprotective agents, stem cell therapy, and advanced rehabilitation modalities. **Results/Findings**: Our review focuses on emerging monitoring techniques, including brain tissue oxygenation, cerebral microdialysis, and multimodal strategies, that provide detailed insights but lack standardized application. Interventions such as anti-inflammatory agents, hypothermia, hyperosmolar therapies, and metabolic suppression show mixed efficacy, with few therapies supported by high-level evidence. Novel agents like erythropoietin and progesterone demonstrate neuroprotective potential in preclinical models but remain inconclusive in clinical trials. Stem cell therapies and extracellular vesicle approaches are promising in early studies. Rehabilitation is expanding with virtual reality, robotics, and neurostimulation to promote neuroplasticity. Personalized medicine approaches incorporating biomarkers and machine learning may refine prognostication and guide therapy. Global inequities persist, particularly in low-resource settings. **Conclusions**: TBI care is shifting toward individualized, multimodal, and technology-driven strategies. While emerging therapies show promise, high-quality randomized trials and global implementation strategies are needed to improve outcomes and reduce disparities.

## 1. Introduction

Traumatic brain injury (TBI) represents a significant global health burden and is a leading contributor to death and disability worldwide, impacting individuals, healthcare systems, and broader societies [1]. In 2021, there were an estimated 20.84 million new cases and 37.93 million prevalent cases of TBI globally, accounting for 5.48 million years lived with disability (YLDs) [2]. Although the absolute numbers of cases have risen substantially since 1990, age-standardized incidence, prevalence, and YLD rates have declined by approximately 20%, reflecting some improvements in prevention and care [2,3,4]. Marked geographic variation exists: the highest age-standardized incidence and prevalence rates were reported in Eastern Europe and Australasia, while the lowest were observed in Western and Eastern Sub-Saharan Africa. Notably, countries with a higher sociodemographic index (SDI) achieved the greatest reductions in age-standardized rates, whereas low-SDI countries showed minimal improvement over the same period [2]. These findings underscore that the global burden of TBI is likely underestimated in low- and middle-income countries, where surveillance systems remain limited and outcomes are disproportionately worse. In the United States, TBI accounted for 214,110 hospitalizations in 2020 and more than 69,000 deaths in 2021, with older adults most affected [1,2,5,6]. Long-term sequelae include cognitive, behavioral, and functional impairments, as well as an elevated risk of epilepsy and neurodegenerative diseases [7,8].

The mechanism of TBI is two-fold: the acute phase, occurring immediately after the injury, and the chronic phase, involving complex cellular and molecular changes manifesting over many years. Thus successful management of TBI requires addressing the challenges associated with each phase; namely, the acute phase treatment focuses on stabilization, minimization of complications, and early recognition of sequelae, whereas the post-acute phase employs rehabilitation and reintegration strategies and addresses mental health concerns, aiming to enhance functional recovery and community engagement [8,9,10]. This review further delves into an overview of TBI, with a focus on advanced diagnostic techniques and developments in treatment modalities to address injury sequelae. We will also discuss different neuroprotective strategies used to prevent secondary brain injuries including pharmacological, non-pharmacological and rehabilitation techniques that aim to improve outcomes in TBI patients.

## 2. Materials and Methods

We conducted a narrative review of the literature on traumatic brain injury (TBI), searching PubMed, MEDLINE, and Google Scholar. Search terms included “traumatic brain injury”, “TBI”, “trauma”, “brain injury”, “head injury”, “concussion”, “epidemiology”, “pathophysiology”, “neuroprotection”, “monitoring”, and “rehabilitation”. Articles published in English were included, with emphasis on randomized trials, meta-analyses, and recent guideline updates. Data were synthesized to summarize epidemiology, mechanisms of injury, monitoring strategies, and current as well as emerging therapeutic interventions.

## 3. Results/Findings

### 3.1. Pathophysiology

The pathophysiology of raised intracranial pressure (ICP) in TBI is complex and involves a number of direct and secondary causes that lead to an increase in volume and pressure in the intracranial vault and consequential decrease in cerebral blood flow [11,12]. The primary insult is a result of the direct mechanical forces or load that sets the head and, by extension, the brain into abnormal directions over a matter of milliseconds [13]. These mechanical forces and biomechanisms dictate the type of injury which can be focal, such as a hematoma, or diffuse in the case of axonal shearing and diffuse axonal injury [13,14,15,16].

**Figure 1:**
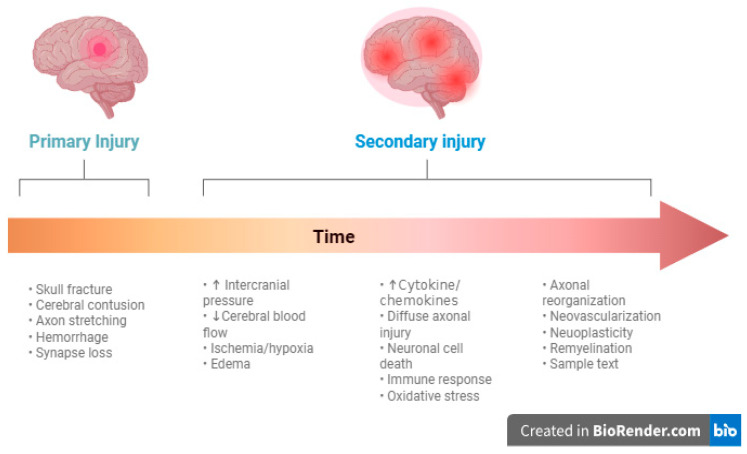
Illustration of the mechanisms of TBI in relation to time. Created in https://BioRender.com.

Secondary mechanisms are a result of a cascade of events that occur after the initial trauma that evolve over an extended period of time. These processes involve different molecular changes such as the increase in glutamate in the extracellular space, formation of oxygen free radicals, activation of kinase cascades and mitochondrial dysfunction [17]. The combined metabolic effects result in brain edema which was classically subdivided into vasogenic edema and cytotoxic edema but is now understood to be more of a continuum. Cytotoxic edema is mediated through a number of intracellular mechanisms that lead to increases in sodium and water. Vasogenic edema relates to disruption of the blood–brain barrier and extravasation of fluid in the extracellular space. When these primary and secondary causes combine to raise intracranial pressure, this ultimately leads to decrease in cerebral perfusion pressure and subsequent neuronal ischemic death [13]. The lasting deficits from this damage can be devastating, and much of the current research focuses on targeting and mediating these inflammatory processes with the goal of improving clinical outcomes. Figure 1 summarizes the mechanisms of TBI.

### 3.2. Advanced Monitoring Techniques

#### 3.2.1. Brain Tissue Oxygenation

Elevated intracranial pressure (ICP) is a common complication in patients with (TBI). Although ICP monitoring is crucial for managing intracranial pressure, it does not necessarily guarantee adequate brain oxygenation even when ICP levels are acceptable. To ensure proper brain oxygenation a more specific and direct method is used [18,19].

Parenchymal brain tissue oxygenation (PbtO_2_) can be measured by inserting an oxygen-sensing probe through a standard burr hole, using CT and MRI guidance, into the frontal lobe white matter [20,21]. Monitoring PbtO_2_ allows clinicians to make modifications to treatment at early signs of hypoxia, commonly defined as PbtO_2_ values below 20 mmHg, and initiate interventions indicated to prevent further damage [21,22,23]. These interventions could include increasing the fraction of inspired oxygen (FiO_2_), increasing cerebral perfusion pressure (CPP), or decreasing ICP [24]. Even increasing FiO2 for as briefly as 15 min has been shown to increase PbtO_2_, although the relationship between arterial partial pressure of oxygen (PaO_2_) and PbtO_2_ can be highly variable [25]. One meta-analysis found that PbtO_2_ monitoring is associated with decreased mortality and ICP compared to ICP/CPP monitoring [26], while another meta-analysis showed benefits on mortality but no impact on functional outcomes [27]. These studies are often limited by a paucity of high-quality reports on the efficacy of these interventions, so further research is still needed in this area.

#### 3.2.2. Microdialysis

Cerebral microdialysis (CMD) is used to monitor small molecular weight substances in brain tissue in order to evaluate for metabolic disturbances [21]. CMD catheters are inserted via a single burr hole into the white matter of the nondominant frontal lobe so that molecules in the brain interstitium are free to diffuse through a semipermeable membrane for collection [28].

The metabolic state of the brain is assessed by analyzing several biomarkers including glucose, glycerol, glutamate, and the lactate-pyruvate ratio (LPR) [28]. Each marker provides insight into the metabolic state, with LPR being of particular interest as it can help diagnose metabolic crisis. Increased LPR indicates either ischemia or mitochondrial dysfunction, with ischemia associated with low pyruvate and low PbtO_2_ while mitochondrial dysfunction is associated with high pyruvate and normal PbtO_2_ [29]. Glutamate is an excitatory transmitter that is released excessively in ischemia that is thought to cause deleterious excitotoxicity, making it a potential prognostic marker [29]. Glucose and glycerol are challenging to interpret, as there is insufficient data to establish an optimal brain glucose range and glycerol is a nonspecific marker of oxidative stress with limited utility in the post-injury state [28,29].

Although CMD provides detailed information about brain chemistry, it is unclear if it results in higher level care. A systematic review found that CMD metabolic markers were associated with functional outcomes 3–6 months post-injury, although it is unclear if there is any association with tissue outcome due to a lack of focused studies [30]. Current literature provides support for the use of CMD to monitor clinical status, although it should be noted that CMD analysis is limited by a focal sample area and definitive consensus on how it correlates with outcomes [30].

#### 3.2.3. Multimodal Monitoring

**Figure 2:**
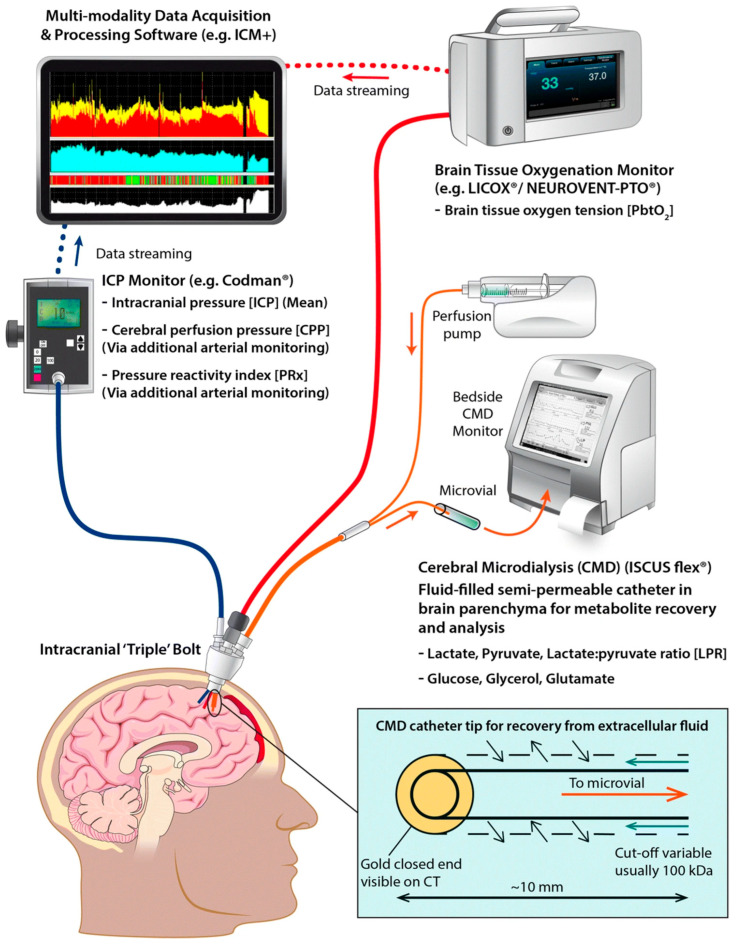
Illustration of invasive multimodal monitoring combining ICP, PbtO_2_, and CMD monitoring. ICP intracranial pressure, PbtO_2_ partial brain tissue oxygen pressure, CMD cerebral microdialysis, PRx pressure reactivity index, CPP cerebral perfusion pressure originally published by Khellaf et al. [36].

Multimodal monitoring is a technique that combines all the previously mentioned modalities by placing multiple devices to monitor ICP, PbtO2, and CMD using a single burr hole at bedside [31]. Figure 2 depicts this method of multimodal data collection. Several studies have investigated the use of multimodal monitoring in patients with TBI. For example, the BOOST2 phase II clinical trial (ClinicalTrials.gov identifier NCT 00974259) found that treatment protocols based on multimodal monitoring with ICP and PbtO_2_ were associated with lower mortality and better outcomes compared to ICP monitoring alone [32,33]. However, the OXY-TC multicenter trial (ClinicalTrials.gov identifier NCT02754063) did not find superiority of multimodal monitoring except for post hoc analysis that suggested some benefit for patients with high ICP on admission [34]. It has been suggested that the difference in conclusions between BOOST2 and OXY-TC may be due to the various methodological differences in the study including frequency of measurements, enrollment criteria, and treatment algorithms [35].

Another observational study of TBI and subarachnoid hemorrhage patients focused on examining how measures including ICP, PbtO_2_, and CMD analysis co-occur found an association between cerebral hypoxia and outcome, consistent with prior studies [19].

While multimodal monitoring could provide clinicians with bountiful data, the lack of techniques’ standardizations across institutions makes it hard to best translate that information into established treatment guidelines. 

### 3.3. Non-Invasive Monitoring

Currently, advanced monitoring requires these invasive techniques that inevitably cause further trauma to an already damaged nervous system. Near-infrared spectroscopy (NIRS) has shown promise in measuring cerebral oxygenation noninvasively during cardiac surgery [37]. NIRS has potential applicability to TBI patients, but the technology needs further study and likely TBI-specific adjustments [38]. Transcranial Doppler ultrasound (TCD) is another non-invasive technique that can be used to evaluate the major cerebral vessels and ventricles via anatomical windows [39]. NIRS and TCD in concert could open up non-invasive monitoring options for neurocritical patients in the future if they can be shown to be accurate and reliable in these populations. 

### 3.4. Therapeutic Interventions

Therapeutic interventions remain limited as much of the TBI treatments employed today lack large, randomized control trials [40]. Indeed, in the Guidelines for the Management of Severe Traumatic Brain Injury published by the Brain Trauma Foundation (BTF) and updated most recently in 2016, only one Level 1 recommendation was published [41]. We recommend that for clinical practice physicians refer to these guidelines for detailed treatment decisions. Currently, treatments largely remain confined to altering basic physiologic parameters such as hemodynamic, respiratory and body temperature in achieving optimal CPP. Within these limitations, clinicians have several methods to modulate these factors which are discussed below.

#### 3.4.1. Blood Pressure Augmentation

Increasing blood pressure has been a mainstay of treatment by means of raising perfusion pressure [42]. However, the cardiovascular complications related to prolonged hypertension are a major concern. For this reason, developing dynamic tools that are able to adapt to each individual patient’s physiology is being actively studied in current research.

#### 3.4.2. Anti-Inflammatory Agents

Neuroinflammation is theorized to be a critical component of secondary injury in TBI patients following the primary insult. Synthetic anti-inflammatory agents, including glucocorticoids and nonsteroidal anti-inflammatory drugs (NSAIDs), modulate various facets of the inflammatory process. These include inhibiting the release of pro-inflammatory cytokines, suppressing microglial activation, modulating signaling pathways, reducing oxidative stress, and attenuating neuroexcitatory neurotransmitters [43]. Additionally, NSAIDs such as aspirin and celecoxib, may provide therapeutic benefits for major depression and schizophrenia, potentially aiding long-term care in TBI patients [44]. Despite their promise in cell-based and pre-clinical models, these anti-inflammatory therapies have not been adopted as standard care for TBI patients, as limited clinical trials have yielded mixed results compared to findings from animal models [43].

A 2023 retrospective cohort study evaluated the use of celecoxib, ibuprofen, and dexamethasone within five days of TBI diagnosis. The study reported a 1-year survival rate of 96.1% in the celecoxib group compared to 93.1% in the non-celecoxib group (*p* < 0.0001). Celecoxib was also associated with reduced gastrostomy dependence, seizure activity, and myocardial infarction (*p* = 0.017, 0.027, and 0.021, respectively) but did not lower the risk of pulmonary embolism, deep vein thrombosis, or stroke. Ibuprofen use improved 1-year survival probability and long-term outcomes, whereas dexamethasone was linked to higher morbidity despite increased 1-year survival. However, the study’s conclusions are limited by the lack of data on dosage, administration route, and treatment duration [45].

#### 3.4.3. Hypocapnia

Hypocapnia is now regarded as a means of lowering ICP for brief periods in the setting of dangerously high ICP. It is now widely accepted that prolonged periods of hypocapnia can be detrimental by causing vasoconstriction. Recent studies have looked at using regional cerebral oxygenation parameters such as brain tissue oxygenation (PbtO2) in guiding ventilation strategies, as a PacO2 threshold may be too simplistic [44].

#### 3.4.4. Hypothermia

Hypothermia in the clinical setting for TBI patients is the controlled lowering of the core body temperature to 32–35 degrees Celsius. This process is suggested to provide neuroprotection in TBI patients by reducing the cerebral metabolic rate of oxygen (CMRO2) and by aiding in the maintenance of the blood–brain barrier (BBB), reducing the release of excitatory neurotransmitters, attenuating the generation and release of free radicals, and decreasing inflammation [46].

Despite its theoretical potential to mitigate secondary injury, hypothermia has shown inconsistent clinical outcomes. The 2018 POLAR-RCT randomized 511 severe TBI patients to hypothermia (33–35 °C) or normothermia (36.5–37.5 °C) within three hours of injury. Hypothermia, maintained for 72 h with gradual rewarming, showed no significant difference in favorable neurological outcomes (48.8% vs. 49.1%; *p* = 0.94) or six-month mortality (21.1% vs. 18.4%; *p* = 0.45) [47]. 

Similarly, the Eurotherm3235 trial, which enrolled 387 TBI patients with elevated intracranial pressure (ICP > 20 mmHg), found less favorable neurological outcomes with hypothermia (25.7% vs. 36.5%; *p* = 0.03) and heightened mortality risk (hazard ratio 1.45; 95% CI 1.01–2.10; *p* = 0.047) [48]. 

Recent meta-analyses and systematic reviews investigating the effect on functional outcomes, mortality, and adverse effects of hypothermia in TBI patients also reflect inconsistent findings. A 2021 review of six RCTs on early prophylactic hypothermia (PH) found no significant differences in improvement of favorable neurological outcomes or reduction in mortality (risk ratio (RR) 1.03, *p* = 0.65; RR = 1.11, *p* = 0.32, respectively), even when patients were subject to variable hypothermia durations and rewarming protocols. Adverse reactions, such as pneumonia, coagulopathies, and hypotension, were observed but not consistently present in all the trials [49]. Likewise, the review in 2019 that included 23 RCTs resulted in variable results, including increased mortality in the therapeutic hypothermia (TH) group (RR 1.26, *p* = 0.020, yet decreased mortality (RR = 0.83, *p* = 0.01) in subgroup analysis when TH was initiated within 24-h post-TBI. A reduced risk of poor outcomes was noted (RR 0.78, *p* = 0.001), while risk of pneumonia was more pronounced in the TH groups (RR 1.48, *p* = 0.007) [50]. The 2024 meta-analysis, which analyzed 32 RCTs, suggested a reduction in poor functional outcomes (RR 0.77, 95% CI 0.67–0.88) and improved mortality (RR 0.81, 95% CI 0.68–0.96) in the pooled analysis. However, in a subgroup analysis, stratified by the cooling method, unfavorable outcomes and mortality in the systemic surface cooling and cranial cooling groups were reduced, but no significant difference in the intravenous or gastric cooling groups was identified [51].

#### 3.4.5. Hyperosmolar Therapies

Hyperosmolar therapies such as mannitol and hypertonic saline (HTS) have been a mainstay of treatment for elevated ICP [52]. The BTF guidelines only reference mannitol due to its long history of clinical use and research supporting positive effects in reducing ICP. Mannitol, unlike hypertonic saline, however, can cause hypovolemia via diuresis which can decrease CPP [53]. Additionally, there have been concerns related to brain edema with repeated use of mannitol. More recent studies have shown a positive trend towards use of hypertonic saline compared to mannitol [54,55], although this has been questioned by others [56]. Additional sugars such as glycerol have shown promise in their dual action of reducing ICP and as a free radical scavenger and antioxidant [55]. It is possible that future guidelines released by the BTF will incorporate HTS in their recommendations and possibly favor it over mannitol.

The use of albumin in TBI management remains controversial. The randomized controlled SAFE-TBI trial found that hypotonic (4%) albumin was associated with higher mortality, resulting in guidelines removing recommendations for albumin in favor of isotonic fluid resuscitation [57]. In contrast, administration of hypertonic (20–25%) albumin at low infusion rates as described in the Lund protocol has been associated with lower mortality rates [57,58]. A meta-analysis redemonstrated this benefit, but interpretation is limited by high risk of bias in existing studies and a lack of prospective trials [58]. Further investigation into the risks and benefits of hypertonic albumin is warranted to clarify and refine current guidelines.

#### 3.4.6. Metabolic Suppression

Suppression of CMRO2 through sedatives such as barbiturates and propofol has been investigated as a means of reducing ICP by reduction in cerebral blood flow [59]. Unfortunately, there are multiple disadvantages related to the use of high dose sedatives including reduction in blood pressure and metabolic derangements [60]. The BTF guidelines state that prophylactic use of sedatives to achieve burst suppression is not recommended [41]. High dose barbiturates can decrease mortality, however, in patients with elevated ICP refractory to medical and surgical treatment [61]. Since the publication of BTF guidelines in 2016, no major RCT has been published on barbiturate therapy. Additionally, an exact treatment pathway and algorithm have not been accepted for refractory elevation in ICP. It is increasingly recognized that the selection of sedatives is important as well as the patient population in which it is used.

#### 3.4.7. Surgical Management

Decompressive craniectomy (DC) is a technique to reduce ICP by exposing the dura mater to the atmosphere [62]. A distinction is made with primary DC in reducing ICP in the early stages of TBI with secondary DC which is used in refractory cases of TBI [63]. The BTF guidelines in 2016 were very specific with regard to the type of craniectomy that is recommended as well as when it should be implemented. There is conflicting data with regard to the short term and long-term effects with decompressive craniectomy. The RESCUEicp trial published shortly after the release of the BTF guidelines in 2016 demonstrated lower mortality rates at the expense of higher rates of vegetative states as well as lower and upper severe disability [64]. There are still considerable questions that remain unanswered in the use of DC. It is possible that future research and guidelines will incorporate patient subtypes who may benefit more than others, and patient outcome must be heavily weighed in the decision to proceed with a craniectomy when discussing this option with patient families.

### 3.5. Emerging Therapies

Preclinical in vitro and in vivo studies suggest that anti-inflammatory agents such as erythropoietin (EPO) and progesterone, as well as stem cell therapies, hold significant neuroprotective potential in TBI.

#### 3.5.1. Erythropoietin

EPO is a glycoprotein hormone that is known to stimulate erythropoiesis and is synthesized in the kidney and the liver. The agent is of particular interest for neuroprotection in TBI patients due to its anti-inflammatory, antioxidant, anti-excitotoxic, and anti-edematous properties [65].

In a 2018 animal study, rats were randomized into three groups: non-TBI, TBI with saline, and TBI with 5000 IU/kg intraperitoneal EPO. The EPO group received therapy for 30 min post-injury and daily for four days. TBI groups were further divided for euthanasia on days 1 or 4. Brain MRI, immunohistofluorescence, immunohistochemistry, and protein analysis revealed that early and repeated EPO administration preserved BBB integrity and reduced cytotoxic brain edema in rat TBI models [66]. 

Despite encouraging findings from TBI models, clinical studies of EPO for TBI have shown limited efficacy. The EPO-TBI trial randomized 606 patients to receive either 40,000 units of subcutaneous EPO or saline within 24 h of injury, followed by weekly doses for up to three weeks. At six months, the primary outcome—reduction in patients with unfavorable Glasgow Outcome Scale-Extended (GOS-E) scores (1–4)—showed no significant difference between the EPO and placebo groups (44% vs. 45%; *p* = 0.9). Secondary outcomes, including mortality and thrombotic events, also revealed no significant differences (mortality: 11% vs. 16%, *p* = 0.07; DVT: 16% vs. 18%, *p* = 0.44) [67]. A median of six-year follow-up of 356 patients further confirmed no improvement in long-term survival (hazard ratio: 0.73, *p* = 0.17) or neurological outcomes (GOS-E: 63% vs. 55%; *p* = 0.14) [68].

Yet, in 2023 a Bayesian network meta-analysis, including 23 trials, analyzed the safety and efficacy of pharmacological neuroprotective therapies in TBI patients. Of the interventions included, seven studies evaluated EPO and demonstrated a reduction in all-cause mortality (RR: 0.68; 95% CrI: 0.50–0.93) as well improved functional recovery (RR: 1.55; 95% CrI: 1.03–2.35), primarily by Glasgow Outcome Scale (GOS), in the EPO group compared to placebo [69].

#### 3.5.2. Progesterone

Progesterone is a steroid hormone that has a major role in the reproductive system and is synthesized in the ovaries, adrenal glands, and the placenta during pregnancy. It acts diffusely throughout the body, and even plays a factor in homeostasis, development, and behavior [70]. Like erythropoietin (EPO), progesterone exhibits anti-inflammatory properties, reduces cellular apoptosis, and mitigates vasogenic edema, positioning it as a potential neuroprotective therapy [71].

In a 2024 mouse TBI model, subjects were divided into progesterone-treated, vehicle-treated, and sham groups. The treatment group received 8 mg/kg of intraperitoneal progesterone 1-h post-injury and continued for three days. Neurological function, assessed via the Motor Water Maze (MWM) test, showed improved spatial memory in the progesterone group, evidenced by reduced latency times compared to controls on days 8–11 (*p* £ 0.027) post-TBI. Progesterone treatment significantly reduced edema (*p* = 0.003), neutrophil infiltration, microglial activation, and several anti-inflammatory markers compared to the TBI control [72]. Results from this study demonstrate the capacity of progesterone to preserve the BBB integrity and improve neurological function, paving the way for progesterone use in clinical studies.

Clinical studies, though limited in quantity, have yet to demonstrate consistent or long-term benefits of progesterone therapy in TBI. The ProTECT III trial, a multicenter phase III study with 882 patients, assessed intravenous (IV) progesterone versus placebo for six-month outcomes using the dichotomized GOS-E. Progesterone treatment, initiated within four hours of injury with a 1-h loading dose followed by a 71-h infusion and 24-h taper, showed no significant differences, leading to trial termination [73]. 

Similarly, the SyNAPSE trial enrolled 1195 patients to evaluate IV progesterone efficacy on six-month neurological outcomes using the GOS. Patients received treatment within eight hours of injury, with a 1-h loading dose (0.71 mg/kg/hr) followed by a 119-h infusion (0.50 mg/kg/hr). At six months, no significant differences were observed, with favorable outcomes reported in 50.4% of the progesterone group and 50.5% of the placebo group (adjusted odds ratio 0.96; 95% CI 0.77–1.18; *p* = 0.88) [71].

In 2019, a meta-analysis assessing the neuroprotective effect of progesterone on severe TBI patients was conducted on eight RCTs. Findings at 3 months post-TBI revealed decreased mortality (RR = 0.59, *p* = 0.001) and improved GOS score, suggesting superior neurological outcomes (RR = 1.51, *p* = 0.007) in the progesterone group compared to the placebo group. However, mortality at 6 months post-TBI did not result in a significant difference between the two groups [74]. In 2021, a systematic review and meta-analysis investigated the effects of progesterone in patients with TBI. After screening a total of 599, 11 studies were included in the review, and the quality of these studies was assessed and used to categorize them. The study concluded that progesterone did not decrease the mortality rate in patients with TBI [75].

Furthermore, another systematic review and meta-analysis on the effect of progesterone on preclinical animal models was subsequently conducted in 2022. A total of 969 studies were initially screened, and 47 were included in their analysis. It concluded that progesterone decreased the adverse effects associated with TBI, such as brain edema and lesion size [76].

Since the ProTECT III and SyNAPSE trials, larger human studies have been limited. Although these phase III trials did not demonstrate significant benefits for progesterone, encouraging findings from animal studies may inform and guide future clinical trials exploring its efficacy as a neuroprotectant in TBI patients.

#### 3.5.3. Stem Cell Therapies

Stem cells are undifferentiated cells that can differentiate into many other functional cell types. These cells have the potential to regenerate and repair damaged tissues [77]. Stem cell therapies, including mesenchymal stem cells (MSCs), extracellular vesicles (EVs), neural stem cells (NSCs), and exosomes, have demonstrated beneficial neurological effects in preclinical studies of TBI models. MSCs, which are multipotent progenitor cells, possess low immunogenicity, high immunosuppressive activity, and can be isolated from multiple sources such as bone marrow, adipose tissues, and birth-related tissues. In 2021, a systematic review and meta-analysis highlighted that MSCs significantly improved sensorimotor and cognitive functions in TBI rodent models, especially when implanted directly into the lesion [78]. EVs, secreted by MSCs, encompass microvesicles and exosomes. As a cell-free therapy, EVs can cross the blood–brain barrier (BBB) and deliver biological components. In preclinical TBI models, EVs have demonstrated the ability to reduce neuronal damage and inflammatory signaling, thereby mitigating cognitive, behavioral, and motor deficits [79,80]. NSCs are multipotent cells that differentiate into neural cells of the nervous system [81]. The PISCES-2 prospective study investigated the safety and impact on upper limb function of subacute-chronic ischemic stroke patients after the stereotactic administration of CTX0E03 human neural stem cells (hNSCs). The primary outcome, defined as a ≥2-point improvement in the Action Research Arm Test (ARAT), was achieved by 1 of 23 patients at 3 months and by 3 patients at 6 and 12 months, spotlighting hNSCs potential to improve motor function [82].

The STEMTRA trials enrolled 61 chronic TBI patients to evaluate the efficacy and safety of stereotactically administered SB623 cells, a modified allogenic bone marrow-derived mesenchymal stem cell (MSC) therapy, at varying doses. The primary outcome, motor recovery, was assessed using the Fugl-Meyer Motor Scale (FMMS). A significant improvement in FMMS scores (≥10 points) was observed in 39.1% of the SB623-treated group compared to 6.87% in the control group (*p* = 0.039) [83].

Encouraging preclinical data, along with findings from stroke and chronic TBI studies, highlight the significant potential of stem cell therapies to enhance outcomes in TBI patients. These results emphasize the need for further research to optimize administration protocols, assess personalized approaches, and investigate the efficacy of combination therapies as neuroprotective strategies for TBI.

### 3.6. Advanced Rehabilitation Techniques

Rehabilitation is essential for facilitating functional recovery and community reintegration in TBI patients. Traditional approaches, including physical, occupational, and speech therapies, remain cornerstone interventions. However, given the complexity of TBI and the unpredictability of the recovery process, which can last years, advanced rehabilitation modalities are being studied [84]. Advanced rehabilitation techniques are technology-driven, designed to promote neuroplasticity, and tailored to improve cognitive and motor recovery. Cognitive therapies, including computer-assisted programs, target improvements in attention, memory, and executive function [85]. Neurofeedback offers another avenue, allowing patients to learn self-regulation skills, thereby increasing attention and improving mental health [86]. Virtual reality (VR) has emerged as a tool for both cognitive and motor recovery, engaging patients in repetitive motor tasks that increase self-awareness and improve decision-making [87]. Robotic-assisted therapies are thought to support the recovery of upper and lower limb functions, as well as gait training, and may play a significant role in TBI rehabilitation [88,89]. Similarly, transcranial stimulation techniques (TCS), including transcranial magnetic stimulation (TMS), enhance neuroplasticity and motor recovery by modulating neural activity through electromagnetic or electric currents targeting brain lesions [90]. While many of these advanced approaches have been studied in stroke rehabilitation, these techniques are now beginning to be explored for cognitive and motor recovery in TBI patients.

### 3.7. Innovative Techniques for TBI Treatment

Much of the focus of research since the publication of the 2016 guidelines in TBI has been individualization of ICP thresholds. The 2016 guidelines recommended treating ICP >22 mmHg given the association with increased mortality above these pressures; however, this threshold was based on a single center retrospective study and is only a level IIb recommendation. Recent observational studies and noncontrolled series have demonstrated variability in ICP treatment threshold ranges, from 15 mmHg to 25 mmHg [91]. More importantly, it is increasingly recognized that strict cutoffs for treatment of ICP is probably too simplistic and more patient specific approaches are needed [92,93].

The pressure reactivity index (PRx), for example, is a tool that looks at the integrity of the cerebral autoregulation curve for each patient [94]. A rise in ICP with increase in MAP indicates a non-intact autoregulation curve and vice versa. A number between −1 and 1 is generated for each patient where a positive value represents a non-intact curve [95]. Studies have demonstrated an association with PRx values and outcomes in patients with TBI [96]. The ideal CPP can be determined for each patient in which the PRx is lowest, and patients whose PRx are kept lowest have been shown to have better outcomes. Other individualized metrics that can be utilized include ICP waveform (ICPW) or pulse morphology as well as invasive and non-invasive techniques [97]. While it is possible that these may be incorporated in future guidelines, no RCT has provided conclusive evidence in favor of one such tool or monitoring technique.

### 3.8. Personalized Medicine in TBI Management

TBI management is often complicated by the broad heterogeneity between individuals, for that reason there has been increased interest in establishing known biomarkers that could be correlated with individual prognosis or targeted by treatment. Biomarkers are downstream products of damage to neurons, axons, glial cells, or other aspects of the nervous system that are typically measured using enzyme-linked immunosorbent assay (ELISA) or mass spectrometry [98]. A pilot multicenter study used machine learning to identify biomarkers that were subsequently found to be associated with unfavorable outcomes and specific genetic polymorphisms [99]. These biomarkers have the potential to offer personalized recommendations and therapeutic options.

Astrocytic glial fibrillary acidic protein (GFAP) is a marker with some of the most robust evidence for its application. GFAP levels have been shown to correlate with brain damage, even being able to identify TBI patients with normal head CT imaging [100]. Combining GFAP with S100, another astrocytic marker, into a predictive model had a high ability to predict outcomes 6 months after injury [101]. The multicenter ALERT-TBI (ClinicalTrials.gov identifier NCT01426919) trial found that a combined assay with GFAP and neuronal ubiquitin C-terminal hydrolase-L1 (UCH-L1) could detect TBI with sensitivity 0.976–1.00, specificity of 0.344–0.364, and negative predictive value of 0.996–1.00 [102]. The TRACK-TBI trial demonstrated that GFAP and UCH-L1 plasma concentration on the day of injury predict death and unfavorable outcome at 6 months, but not incomplete recovery (ClinicalTrials.gov identifier NCT02119182) [103].

An advantage of many of these biomarkers is that they can be detected using blood tests, as opposed to more invasive analyses of CSF. There are no specific therapies for TBI, so further research into this area is needed to investigate if targeting these biomarkers could represent disease-modifying treatment for these patients. Current barriers to implementation include a lack of standardized assays and insufficient clinical data to decisively conclude the significance of any biomarkers of interest [104].

## 4. Challenges and Controversies

### 4.1. Variability in Guideline Implementation

The BTF guidelines remain the staple for the treatment of patients with traumatic brain injury for the majority of centers providing neurocritical care. Given the low quality of evidence in much of the recommendations, there is significant variability, however, in its implementation. In one survey of European centers, 75% of respondents reported using the BTF guidelines while 17% used no guidelines and the remaining 8% other guidelines [105]. In a systematic review of guidelines adherence, 22 studies analyzed reported a wide variability in guideline adherence (18–100%) with adherence dependent on strength of evidence and invasiveness of the recommendation [106].

The importance of implementation of guidelines has been increasingly studied. Indeed, multiple studies have reported that the implementation of the BTF guidelines can reduce poor outcomes and even mortality [107,108]. A recent systematic review found an association between the adherence to guidelines and reduced mortality, but the authors felt that this could only be interpreted as being preliminary based on other confounding variables [106].

### 4.2. Balancing Benefits and Risks

Much of the emphasis in the treatment of TBI has been on the reduction in ICP and optimizing CPP. In the last decade, however, more attention is being paid to other measures such as pressure autoregulation, tissue metabolic requirements and brain tissue oxygenation [109]. It is possible that many patients are both overtreated and undertreated in their TBI management by hyperfocusing on ICP and CPP. With the use of dynamic and multimodal monitoring in the future, we may have better knowledge of who to more aggressively treat ICP and what type of therapies to employ, allowing for more precise treatment of TBI.

### 4.3. Ethical Dilemmas in Invasive Monitoring and Experimental Therapies

Ethical considerations in the study and treatment of TBI remain an important factor in TBI treatment protocols. The issue of consent in patients who cannot communicate their desires and understand risk and benefit will always be a significant issue [110]. Studies that look at the use of invasive monitoring or require invasive monitoring as part of measurement of various endpoints may expose patients to unnecessary procedures and harm [111].

The clinical equipoise of research trials and the use of patients from low-resource countries in studying certain therapies must be at the forefront of researchers minds when studying TBI [112]. For example, the BEST TRIP trial looked at the use of ICP monitoring in severe TBI in two South American countries [113]. Although the study had questionable relevance to the host countries [112], it provided valuable evidence to Western nations in which ICP monitoring is available. 

In addition, as therapies improve, clinicians must consider the possibility that TBI treatments may improve survival at the expense of higher morbidity. This has serious consequences considering the possibility of outcomes. The most important example relates to decompressive craniectomy and the possibility that patients may survive due to treatment at the expense of being in persistent vegetative states [114]. As of this time, no recommendations have been made on this matter, but it is possible that there will be more attention on this topic as treatment methods improve.

### 4.4. Gaps in Evidence

There remain some significant gaps in the treatment and management of TBI. As discussed previously, individualization of ICP thresholds remains a hot topic in TBI research. Further areas of clarification include the selection of hyperosmolar therapies. Indications and use of craniectomy remains a topic that requires more evidence to determine which patients may benefit versus those who are expected to suffer high morbidity. Better evidence is needed to support ICP monitoring. Additional data is needed in transfusion medicine in determining the optimal thresholds to administer blood products. Finally, the use of reversal agents and blood products to counteract the effects of antiplatelets and anticoagulants will be an area with new recommendations as novel reversal agents are released into practice and are more widely available.

## 5. Prevention

Prevention of traumatic brain injury (TBI) is an essential public health priority, as reducing the incidence of TBI is far more effective than treating its long-term consequences [2] A public health approach emphasizes both primary prevention, aimed at avoiding injury, and secondary prevention, aimed at mitigating severity once injury occurs. Evidence-based strategies include strengthening road safety through seat belt and helmet laws, improvements in road infrastructure, and enforcement of traffic safety regulations, all of which have demonstrated reductions in TBI incidence [115]. Programs such as the CDC’s *HeadsUp* initiative [115,116,117], which raises awareness among athletes, parents, and coaches, and the *STEADI* program [118], designed to reduce fall-related injuries in older adults, have been shown to effectively lower risk in vulnerable populations [119,120]. International experiences, such as mandatory helmet laws in Vietnam [121], further highlight the profound impact of preventive legislation, with significant reductions in motorcycle-related TBIs and associated healthcare costs. Collectively, this underscore the need for comprehensive prevention programs targeting motor vehicle safety, sports-related injuries, and fall prevention represent some of the most effective strategies to reduce the global burden of TBI [122,123].

## 6. Future Directions in TBI Management

### 6.1. Integration of Artificial Intelligence and Big Data

Clinicians are now able to collect sophisticated and in-depth information about TBI patients with the use of modern technology, and there is now an increasing focus on proper interpretation. An ongoing challenge in neurocritical care is reviewing large volumes of real-time data such as ICP, CPP, and PbtO_2_ and integrating them into a comprehensive care plan. This work can be difficult and time-intensive, so there is interest in developing machine learning technology to assist with outcome prediction and treatment optimization. A machine learning program trained on retrospective ICU data was able to predict episodes of intracranial hypertension (defined as median ICP > 15) in the following 30 min with 92% accuracy when applied to an external validation dataset [124,125]. Another study created an algorithm to analyze ICP waveforms using continuous ICP monitoring data from non-traumatic intracranial hypertension evaluations that predicted ICP elevation >20 within the next 20 min with 97% specificity [126]. The ability to accurately predict changes in ICP would allow treatment of ICP to be proactive and prevent episodes of elevation as opposed to purely reactive treatment. 

Another potential application of machine learning is to help clinicians anticipate likely patient outcomes. One study investigated how machine learning enhanced interpretation of functional magnetic resonance imaging could predict functional deficits of TBI based on the modeled network disruptions [127]. Another study created simple algorithms requiring only three or four variables with machine learning that were demonstrated to predict mortality with 81–84% accuracy [128]. One of these algorithms was retrained and externally validated on an international cohort of 686 patients with resultant accuracy of 76–90% across cohorts [129].

A meta-analysis of machine learning algorithms showed superiority over traditional multivariate regression models with accuracy consistently greater than 80% [130]. Despite the promise of these advances, the integration of artificial intelligence into clinical practice demands a great deal of stewardship. Factors such as biased source data and poor use of statistical analysis can result in algorithms producing conclusions that are wholly incorrect or perhaps only apply to specific subgroups [131].

### 6.2. Global Initiatives

TBI represents a significant global injury burden, with indications that incidence is increasing with population growth [132]. These injuries are both relatively common and can require advanced interventions for proper treatment, raising concern for systematic inadequate treatment of resource-limited regions. It has been shown that the degree of TBI recovery is influenced by a host of socioeconomic factors including race, insurance status, and whether patients live in a rural or urban area [133]. When brought to a global scale, it is practically inevitable that there are vast portions of the population that are not able to access the necessary acute care. For example, rural locations with limited infrastructure often lack robust emergency medical transport.

The Lancet Neurology Commission in 2017 brought attention to the global health problem of TBI and recognized a need for international consensus on definitions and rigorous epidemiological studies, particularly in low- and middle-income countries (LMIC) [134]. A number of major organization bodies have taken an interest in addressing this public health issue. A significant cause of TBIs in LMICs is motor vehicle accidents, which potentially may see improvement from the World Health Organization’s (WHO) “Decade of Road Safety” declared in 2021 [9,132]. China has a notable TBI burden with remarkable advancements over the past few decades, likely attributable to increased access to specific training, improvement to prehospital infrastructure, and dissemination of modern imaging techniques [135]. Many of the challenges addressed by these reforms are generalizable to the majority of other countries. Sufficient access to technology and expertise is often a limiting factor, particularly in remote or rural settings.

Another approach under study is developing techniques within LMICs that work within the existing infrastructure. Integration of machine-learning techniques is primarily being studied in high-income nations at this time, leading to concerns that these emerging tools may not be generalizable to other countries. Prediction models trained on local data from low- and middle-income countries can anticipate clinically relevant outcomes including mortality and length of hospital stay [136]. Decompressive craniotomy is a relatively common procedure in LMICs with some benefit for survival, however current studies are limited so it is difficult to make a conclusive statement on its use [137]. Combining decompressive craniotomy with cisternostomy is a technique that originated in LMICs to treat severe cases of TBI and has been shown to reduce mechanical ventilation and ICU times [138].

Looking to the future, there are many areas that need further investment. One such area is addressing health inequities, for example, by strengthening medical access and transport in rural areas. The National Highways Authority India and The World Bank Road Safety Project in Bangladesh are both initiatives with a focus on providing higher quality emergency care along major highways [9]. Time is of utmost importance in critical brain injuries, so improving this factor has a large potential to improve outcomes. Another priority is international collaboration on clinical trials. Trials are often limited to a single country due to logistical hurdles, but this has resulted in a dearth of internationally accepted standards for definitions and treatment. There are dramatic differences in TBI burden and treatment across nations, but researchers can only speculate about the underlying reasons for those differences [132]. Strengthening global ties in this way could provide valuable insight into both prevention and treatment of TBI.

## 7. Conclusions

Traumatic brain injury remains a major global health challenge with significant personal, societal, and economic consequences. Despite advances in monitoring, rehabilitation, and experimental therapies, there is still no definitive cure, and current management largely focuses on supportive care and mitigating secondary injury. Novel pharmacologic agents, stem cell-based interventions, and technology-driven rehabilitation approaches show promise, but their translation into routine clinical practice requires robust clinical trials and standardized protocols. Equally important are efforts to individualize care through biomarkers, multimodal monitoring, and artificial intelligence-assisted decision-making. Addressing disparities in access to care, particularly in low- and middle-income countries, will be essential to improving outcomes worldwide. Continued multidisciplinary collaboration and global research initiatives are critical for transforming TBI management from reactive stabilization to proactive, personalized, and restorative care.

## Abbreviations

The following abbreviations are used in this manuscript:

TBI	Traumatic brain injury
EPO	Erythropoietin
WHO	World Health Organization
LMIC	Low- and Middle-income countries
GFAP	Glial fibrillary acidic protein
UCH-L1	Ubiquitin C-terminal hydrolase-L1
ELISA	Enzyme-linked immunosorbent assay
ICPW	ICP waveform
TCS	Transcranial stimulation techniques
TMS	Transcranial magnetic stimulation
MSC	Mesenchymal stem cell
FMMS	Fugl-Meyer Motor Scale
hNSCs	Human neural stem cells
ARAT	Action Research Arm Test
MWM	Motor Water Maze
GOS-E	Glasgow Outcome Scale-Extended
DC	Decompressive craniectomy
BTF	Brain Trauma Foundation
LPR	Lactate-pyruvate ratio
CMD	Cerebral microdialysis

## References

[1] CDC Facts About TBI. https://www.cdc.gov/traumatic-brain-injury/data-research/facts-stats/index.html.

[2] Zhong H., Feng Y., Shen J., Rao T., Dai H., Zhong W., Zhao G. (2025). Global burden of traumatic brain injury in 204 countries and territories from 1990 to 2021. Am. J. Prev. Med..

[3] Guan B., Anderson D.B., Chen L., Feng S., Zhou H. (2023). Global, regional and national burden of traumatic brain injury and spinal cord injury, 1990–2019: A systematic analysis for the Global Burden of Disease Study 2019. BMJ Open.

[4] Huang X.F., Ma S.F., Jiang X.H., Song R.J., Li M., Zhang J., Sun T.J., Hu Q., Wang W.R., Yu A.Y. (2024). Causes and global, regional, and national burdens of traumatic brain injury from 1990 to 2019. Chin. J. Traumatol..

[5] CDC TBI Data. https://www.cdc.gov/traumatic-brain-injury/data-research/index.html.

[6] Peterson C., Miller G.F., Barnett S.B.L., Florence C. (2021). Economic Cost of Injury—United States, 2019. MMWR Morb. Mortal. Wkly. Rep..

[7] CDC About Potential Effects of a Moderate or Severe TBI. https://www.cdc.gov/traumatic-brain-injury/about/potential-effects.html.

[8] Dams-O’Connor K., Juengst S.B., Bogner J., Chiaravalloti N.D., Corrigan J.D., Giacino J.T., Harrison-Felix C.L., Hoffman J.M., Ketchum J.M., Lequerica A.H. (2023). Traumatic brain injury as a chronic disease: Insights from the United States Traumatic Brain Injury Model Systems Research Program. Lancet Neurol..

[9] Maas A.I.R., Menon D.K., Manley G.T., Abrams M., Åkerlund C., Andelic N., Aries M., Bashford T., Bell M.J., Bodien Y.G. (2022). Traumatic brain injury: Progress and challenges in prevention, clinical care, and research. Lancet Neurol..

[10] Matney C., Bowman K., Berwick D., National Academies of Sciences, Engineering, and Medicine, Health and Medicine Division, Board on Health Care Services, Board on Health Sciences Policy, Committee on Accelerating Progress in Traumatic Brain Injury Research and Care (2022). Traumatic Brain Injury: A Roadmap for Accelerating Progress.

[11] Leinonen V., Vanninen R., Rauramaa T. (2017). Raised intracranial pressure and brain edema. Handb. Clin. Neurol..

[12] Canac N., Jalaleddini K., Thorpe S.G., Thibeault C.M., Hamilton R.B. (2020). Review: Pathophysiology of intracranial hypertension and noninvasive intracranial pressure monitoring. Fluids Barriers CNS.

[13] White H., Venkatesh B. (2008). Cerebral perfusion pressure in neurotrauma: A review. Anesth. Analg..

[14] Greve M.W., Zink B.J. (2009). Pathophysiology of traumatic brain injury. Mt. Sinai J. Med..

[15] Werner C., Engelhard K. (2007). Pathophysiology of traumatic brain injury. Br. J. Anaesth..

[16] Nakagawa A., Manley G.T., Gean A.D., Ohtani K., Armonda R., Tsukamoto A., Yamamoto H., Takayama K., Tominaga T. (2011). Mechanisms of primary blast-induced traumatic brain injury: Insights from shock-wave research. J. Neurotrauma.

[17] Ladak A.A., Enam S.A., Ibrahim M.T. (2019). A Review of the Molecular Mechanisms of Traumatic Brain Injury. World Neurosurg..

[18] Chesnut R., Aguilera S., Buki A., Bulger E., Citerio G., Cooper D.J., Arrastia R.D., Diringer M., Figaji A., Gao G. (2020). A management algorithm for adult patients with both brain oxygen and intracranial pressure monitoring: The Seattle International Severe Traumatic Brain Injury Consensus Conference (SIBICC). Intensive Care Med..

[19] Lund A., Madsen A.F., Capion T., Jensen H.R., Forsse A., Hauerberg J., Sigurðsson S., Mathiesen T.I., Møller K., Olsen M.H. (2024). Brain hypoxia and metabolic crisis are common in patients with acute brain injury despite a normal intracranial pressure. Sci. Rep..

[20] Häni L., Ropelato M.D., Wagner F., Nowacki A., Söll N., Haenggi M., Raabe A., Z’Graggen W.J. (2021). Individualized Brain Tissue Oxygen-Monitoring Probe Placement Helps to Guide Therapy and Optimizes Outcome in Neurocritical Care. Neurocrit. Care.

[21] Meyfroidt G., Bouzat P., Casaer M.P., Chesnut R., Hamada S.R., Helbok R., Hutchinson P., Maas A.I.R., Manley G., Menon D.K. (2022). Management of moderate to severe traumatic brain injury: An update for the intensivist. Intensive Care Med..

[22] Robinson C.P. (2021). Moderate and Severe Traumatic Brain Injury. Continuum.

[23] Hirschi R., Hawryluk G.W.J., Nielson J.L., Huie J.R., Zimmermann L.L., Saigal R., Ding Q., Ferguson A.R., Manley G. (2019). Analysis of high-frequency PbtO2 measures in traumatic brain injury: Insights into the treatment threshold. J. Neurosurg..

[24] Lin C.M., Lin M.C., Huang S.J., Chang C.K., Chao D.P., Lui T.N., Ma H.I., Liu M.Y., Chung W.Y., Shih Y.H. (2015). A Prospective Randomized Study of Brain Tissue Oxygen Pressure-Guided Management in Moderate and Severe Traumatic Brain Injury Patients. Biomed. Res. Int..

[25] Figaji A.A., Zwane E., Graham Fieggen A., Argent A.C., Le Roux P.D., Peter J.C. (2010). The effect of increased inspired fraction of oxygen on brain tissue oxygen tension in children with severe traumatic brain injury. Neurocrit. Care.

[26] Shen Y., Wen D., Liang Z., Wan L., Jiang Q., He H., He M. (2024). Brain tissue oxygen partial pressure monitoring and prognosis of patients with traumatic brain injury: A meta-analysis. Neurosurg. Rev..

[27] Hays L.M.C., Udy A., Adamides A.A., Anstey J.R., Bailey M., Bellapart J., Byrne K., Cheng A., Jamie Cooper D., Drummond K.J. (2022). Effects of brain tissue oxygen (PbtO(2)) guided management on patient outcomes following severe traumatic brain injury: A systematic review and meta-analysis. J. Clin. Neurosci..

[28] Stovell M.G., Helmy A., Thelin E.P., Jalloh I., Hutchinson P.J., Carpenter K.L.H. (2023). An overview of clinical cerebral microdialysis in acute brain injury. Front. Neurol..

[29] Hutchinson P.J., Jalloh I., Helmy A., Carpenter K.L., Rostami E., Bellander B.M., Boutelle M.G., Chen J.W., Claassen J., Dahyot-Fizelier C. (2015). Consensus statement from the 2014 International Microdialysis Forum. Intensive Care Med..

[30] Zeiler F.A., Thelin E.P., Helmy A., Czosnyka M., Hutchinson P.J.A., Menon D.K. (2017). A systematic review of cerebral microdialysis and outcomes in TBI: Relationships to patient functional outcome, neurophysiologic measures, and tissue outcome. Acta Neurochir..

[31] Foreman B., Ngwenya L.B., Stoddard E., Hinzman J.M., Andaluz N., Hartings J.A. (2018). Safety and Reliability of Bedside, Single Burr Hole Technique for Intracranial Multimodality Monitoring in Severe Traumatic Brain Injury. Neurocrit. Care.

[32] Okonkwo D.O., Shutter L.A., Moore C., Temkin N.R., Puccio A.M., Madden C.J., Andaluz N., Chesnut R.M., Bullock M.R., Grant G.A. (2017). Brain Oxygen Optimization in Severe Traumatic Brain Injury Phase-II: A Phase II Randomized Trial. Crit. Care Med..

[33] Bernard F., Barsan W., Diaz-Arrastia R., Merck L.H., Yeatts S., Shutter L.A. (2022). Brain Oxygen Optimization in Severe Traumatic Brain Injury (BOOST-3): A multicentre, randomised, blinded-endpoint, comparative effectiveness study of brain tissue oxygen and intracranial pressure monitoring versus intracranial pressure alone. BMJ Open.

[34] Payen J.F., Launey Y., Chabanne R., Gay S., Francony G., Gergele L., Vega E., Montcriol A., Couret D., Cottenceau V. (2023). Intracranial pressure monitoring with and without brain tissue oxygen pressure monitoring for severe traumatic brain injury in France (OXY-TC): An open-label, randomised controlled superiority trial. Lancet Neurol..

[35] Diaz-Arrastia R., Bernard F., Shutter L., Barsan W., Silbergleit R. (2024). Monitoring patients with severe traumatic brain injury. Lancet Neurol..

[36] Khellaf A., Khan D.Z., Helmy A. (2019). Recent advances in traumatic brain injury. J. Neurol..

[37] Ali J., Cody J., Maldonado Y., Ramakrishna H. (2022). Near-Infrared Spectroscopy (NIRS) for Cerebral and Tissue Oximetry: Analysis of Evolving Applications. J. Cardiothorac. Vasc. Anesth..

[38] Maas A.I., Citerio G. (2010). Noninvasive monitoring of cerebral oxygenation in traumatic brain injury: A mix of doubts and hope. Intensive Care Med..

[39] Blanco P., Abdo-Cuza A. (2018). Transcranial Doppler ultrasound in neurocritical care. J. Ultrasound.

[40] Buccilli B., Alan A., Baha A., Shahzad A., Almealawy Y.F., Chisvo N.S., Ennabe M., Weinand M. (2024). Neuroprotection strategies in traumatic brain injury: Studying the effectiveness of different clinical approaches. Surg. Neurol. Int..

[41] Carney N., Totten A.M., O’Reilly C., Ullman J.S., Hawryluk G.W., Bell M.J., Bratton S.L., Chesnut R., Harris O.A., Kissoon N. (2017). Guidelines for the Management of Severe Traumatic Brain Injury, Fourth Edition. Neurosurgery.

[42] Vella M.A., Crandall M., Patel M.B. (2017). Acute management of traumatic brain injury. Surg. Clin. N. Am..

[43] Kalra S., Malik R., Singh G., Bhatia S., Al-Harrasi A., Mohan S., Albratty M., Albarrati A., Tambuwala M.M. (2022). Pathogenesis and management of traumatic brain injury (TBI): Role of neuroinflammation and anti-inflammatory drugs. Inflammopharmacology.

[44] Müller N. (2019). COX-2 Inhibitors, Aspirin, and Other Potential Anti-Inflammatory Treatments for Psychiatric Disorders. Front. Psychiatry.

[45] Bhanja D., Hallan D.R., Staub J., Rizk E., Zacko J.C. (2024). Early Celecoxib use in Patients with Traumatic Brain Injury. Neurocrit. Care.

[46] Docherty A., Emelifeonwu J., Andrews P.J.D. (2018). Hypothermia After Traumatic Brain Injury. JAMA.

[47] Cooper D.J., Nichol A.D., Bailey M., Bernard S., Cameron P.A., Pili-Floury S., Forbes A., Gantner D., Higgins A.M., Huet O. (2018). Effect of Early Sustained Prophylactic Hypothermia on Neurologic Outcomes Among Patients with Severe Traumatic Brain Injury: The POLAR Randomized Clinical Trial. JAMA.

[48] Andrews P.J., Sinclair H.L., Rodriguez A., Harris B.A., Battison C.G., Rhodes J.K., Murray G.D. (2015). Hypothermia for Intracranial Hypertension after Traumatic Brain Injury. N. Engl. J. Med..

[49] Wu X., Tao Y., Marsons L., Dee P., Yu D., Guan Y., Zhou X. (2021). The effectiveness of early prophylactic hypothermia in adult patients with traumatic brain injury: A systematic review and meta-analysis. Aust. Crit. Care.

[50] Chen H., Wu F., Yang P., Shao J., Chen Q., Zheng R. (2019). A meta-analysis of the effects of therapeutic hypothermia in adult patients with traumatic brain injury. Crit. Care.

[51] Martyniuk A., Hart S., Lannon M., Mastrolonardo A., Kabbani A., Hafeez D.A., Engels P.T., Sharma S. (2024). Therapeutic Hypothermia Compared with Normothermia in Adults with Traumatic Brain Injury; Functional Outcome, Mortality, and Adverse Effects: A Systematic Review and Meta-Analysis. Neurocrit. Care.

[52] Gharizadeh N., Ghojazadeh M., Naseri A., Dolati S., Tarighat F., Soleimanpour H. (2022). Hypertonic saline for traumatic brain injury: A systematic review and meta-analysis. Eur. J. Med. Res..

[53] Kim J.H., Jeong H., Choo Y.H., Kim M., Ha E.J., Oh J., Shim Y., Kim S.B., Jung H.G., Park S.H. (2023). Optimizing Mannitol Use in Managing Increased Intracranial Pressure: A Comprehensive Review of Recent Research and Clinical Experiences. Korean J. Neurotrauma.

[54] Boone M.D., Oren-Grinberg A., Robinson T.M., Chen C.C., Kasper E.M. (2015). Mannitol or hypertonic saline in the setting of traumatic brain injury: What have we learned?. Surg. Neurol. Int..

[55] Patil H., Gupta R. (2019). A Comparative Study of Bolus Dose of Hypertonic Saline, Mannitol, and Mannitol Plus Glycerol Combination in Patients with Severe Traumatic Brain Injury. World Neurosurg..

[56] Bernhardt K., McClune W., Rowland M.J., Shah A. (2024). Hypertonic Saline Versus Other Intracranial-Pressure-Lowering Agents for Patients with Acute Traumatic Brain Injury: A Systematic Review and Meta-analysis. Neurocrit. Care.

[57] Vincent J.L., Ferrer R., Taccone F.S., Wiedermann C.J., Reinstrup P. (2025). Re-evaluating albumin use in traumatic brain injury. J. Intensive Care.

[58] Wiedermann C.J. (2022). Use of Hyperoncotic Human Albumin Solution in Severe Traumatic Brain Injury Revisited—A Narrative Review and Meta-Analysis. J. Clin. Med..

[59] Choo Y.H., Seo Y., Oh H.J. (2023). Deep Sedation in Traumatic Brain Injury Patients. Korean J. Neurotrauma.

[60] Roberts I., Sydenham E. (2012). Barbiturates for acute traumatic brain injury. Cochrane Database Syst. Rev..

[61] Eisenberg H.M., Frankowski R.F., Contant C.F., Marshall L.F., Walker M.D. (1988). High-dose barbiturate control of elevated intracranial pressure in patients with severe head injury. J. Neurosurg..

[62] Tomita H., Kimura K., Ono Y., Yamada O., Yagihara T., Echigo S. (2001). Life-threatening pulmonary edema following unilateral stent implantation for bilateral branch pulmonary stenosis: Recovery after contralateral stent implantation. Jpn. Circ. J..

[63] Bor-Seng-Shu E., Figueiredo E.G., Amorim R.L., Teixeira M.J., Valbuza J.S., de Oliveira M.M., Panerai R.B. (2012). Decompressive craniectomy: A meta-analysis of influences on intracranial pressure and cerebral perfusion pressure in the treatment of traumatic brain injury. J. Neurosurg..

[64] Hutchinson P.J., Kolias A.G., Timofeev I.S., Corteen E.A., Czosnyka M., Timothy J., Anderson I., Bulters D.O., Belli A., Eynon C.A. (2016). Trial of Decompressive Craniectomy for Traumatic Intracranial Hypertension. N. Engl. J. Med..

[65] Ponce L.L., Navarro J.C., Ahmed O., Robertson C.S. (2013). Erythropoietin neuroprotection with traumatic brain injury. Pathophysiology.

[66] Blixt J., Gunnarson E., Wanecek M. (2018). Erythropoietin Attenuates the Brain Edema Response after Experimental Traumatic Brain Injury. J. Neurotrauma.

[67] Nichol A., French C., Little L., Haddad S., Presneill J., Arabi Y., Bailey M., Cooper D.J., Duranteau J., Huet O. (2015). Erythropoietin in traumatic brain injury (EPO-TBI): A double-blind randomised controlled trial. Lancet.

[68] Skrifvars M.B., Luethi N., Bailey M., French C., Nichol A., Trapani T., McArthur C., Arabi Y.M., Bendel S., Cooper D.J. (2023). The effect of recombinant erythropoietin on long-term outcome after moderate-to-severe traumatic brain injury. Intensive Care Med..

[69] Wang X., Li X., Ma L., Chen H., You C. (2023). Pharmacological components with neuroprotective effects in the management of traumatic brain injury: Evidence from network meta-analysis. Neurol. Sci..

[70] Tetel M.J., Lange C.A., Pfaff D.W., Arnold A.P., Etgen A.M., Fahrbach S.E., Rubin R.T. (2009). 44—Molecular Genomics of Progestin Actions. Hormones, Brain and Behavior (Second Edition).

[71] Skolnick B.E., Maas A.I., Narayan R.K., van der Hoop R.G., MacAllister T., Ward J.D., Nelson N.R., Stocchetti N. (2014). A clinical trial of progesterone for severe traumatic brain injury. N. Engl. J. Med..

[72] Zhou Z., Li Y., Peng R., Shi M., Gao W., Lei P., Zhang J. (2024). Progesterone induces neuroprotection associated with immune/inflammatory modulation in experimental traumatic brain injury. Neuroreport.

[73] Stein D.G. (2015). Embracing failure: What the Phase III progesterone studies can teach about TBI clinical trials. Brain Inj..

[74] Pan Z.Y., Zhao Y.H., Huang W.H., Xiao Z.Z., Li Z.Q. (2019). Effect of progesterone administration on the prognosis of patients with severe traumatic brain injury: A meta-analysis of randomized clinical trials. Drug Des. Dev. Ther..

[75] Bazgir R., Siahposht-Khachaki A., Akbari E., Farzin D. (2021). A study of the therapeutic effects of progesterone in patients with traumatic brain injury: A systematic review and meta-analysis. Arch. Trauma Res..

[76] Nasre-Nasser R.G., Severo M.M.R., Pires G.N., Hort M.A., Arbo B.D. (2022). Effects of Progesterone on Preclinical Animal Models of Traumatic Brain Injury: Systematic Review and Meta-analysis. Mol. Neurobiol..

[77] Biehl J.K., Russell B. (2009). Introduction to stem cell therapy. J. Cardiovasc. Nurs..

[78] Pischiutta F., Caruso E., Lugo A., Cavaleiro H., Stocchetti N., Citerio G., Salgado A., Gallus S., Zanier E.R. (2021). Systematic review and meta-analysis of preclinical studies testing mesenchymal stromal cells for traumatic brain injury. NPJ Regen. Med..

[79] Cai J., Wu J., Wang J., Li Y., Hu X., Luo S., Xiang D. (2020). Extracellular vesicles derived from different sources of mesenchymal stem cells: Therapeutic effects and translational potential. Cell Biosci..

[80] Reed S.L., Escayg A. (2021). Extracellular vesicles in the treatment of neurological disorders. Neurobiol. Dis..

[81] Reis C., Gospodarev V., Reis H., Wilkinson M., Gaio J., Araujo C., Chen S., Zhang J.H. (2017). Traumatic Brain Injury and Stem Cell: Pathophysiology and Update on Recent Treatment Modalities. Stem Cells Int..

[82] Muir K.W., Bulters D., Willmot M., Sprigg N., Dixit A., Ward N., Tyrrell P., Majid A., Dunn L., Bath P. (2020). Intracerebral implantation of human neural stem cells and motor recovery after stroke: Multicentre prospective single-arm study (PISCES-2). J. Neurol. Neurosurg. Psychiatry.

[83] Kawabori M., Weintraub A.H., Imai H., Zinkevych I., McAllister P., Steinberg G.K., Frishberg B.M., Yasuhara T., Chen J.W., Cramer S.C. (2021). Cell Therapy for Chronic TBI. Neurology.

[84] Marklund N., Bellander B.M., Godbolt A.K., Levin H., McCrory P., Thelin E.P. (2019). Treatments and rehabilitation in the acute and chronic state of traumatic brain injury. J. Intern. Med..

[85] Maggio M.G., De Bartolo D., Calabrò R.S., Ciancarelli I., Cerasa A., Tonin P., Di Iulio F., Paolucci S., Antonucci G., Morone G. (2023). Computer-assisted cognitive rehabilitation in neurological patients: State-of-art and future perspectives. Front. Neurol..

[86] Gray S.N. (2017). An Overview of the Use of Neurofeedback Biofeedback for the Treatment of Symptoms of Traumatic Brain Injury in Military and Civilian Populations. Med. Acupunct..

[87] Calderone A., Carta D., Cardile D., Quartarone A., Rifici C., Calabrò R.S., Corallo F. (2023). Use of Virtual Reality in Patients with Acquired Brain Injury: A Systematic Review. J. Clin. Med..

[88] Karunakaran K.K., Pamula S.D., Bach C.P., Legelen E., Saleh S., Nolan K.J. (2023). Lower extremity robotic exoskeleton devices for overground ambulation recovery in acquired brain injury—A review. Front. Neurorobot..

[89] Gassert R., Dietz V. (2018). Rehabilitation robots for the treatment of sensorimotor deficits: A neurophysiological perspective. J. Neuroeng. Rehabil..

[90] Clayton E., Kinley-Cooper S.K., Weber R.A., Adkins D.L. (2016). Brain stimulation: Neuromodulation as a potential treatment for motor recovery following traumatic brain injury. Brain Res..

[91] Stein K.Y., Froese L., Gomez A., Sainbhi A.S., Vakitbilir N., Ibrahim Y., Zeiler F.A. (2023). Intracranial Pressure Monitoring and Treatment Thresholds in Acute Neural Injury: A Narrative Review of the Historical Achievements, Current State, and Future Perspectives. Neurotrauma Rep..

[92] Chesnut R.M., Videtta W. (2020). Situational Intracranial Pressure Management: An Argument Against a Fixed Treatment Threshold. Crit. Care Med..

[93] Zeiler F.A., Ercole A., Cabeleira M., Beqiri E., Zoerle T., Carbonara M., Stocchetti N., Menon D.K., Lazaridis C., Smielewski P. (2021). Patient-specific ICP Epidemiologic Thresholds in Adult Traumatic Brain Injury: A CENTER-TBI Validation Study. J. Neurosurg. Anesthesiol..

[94] Czosnyka M., Czosnyka Z., Smielewski P. (2017). Pressure reactivity index: Journey through the past 20 years. Acta Neurochir..

[95] Tsigaras Z.A., Weeden M., McNamara R., Jeffcote T., Udy A.A. (2023). The pressure reactivity index as a measure of cerebral autoregulation and its application in traumatic brain injury management. Crit. Care Resusc..

[96] Sánchez-Porras R., Santos E., Czosnyka M., Zheng Z., Unterberg A.W., Sakowitz O.W. (2012). ‘Long’ pressure reactivity index (L-PRx) as a measure of autoregulation correlates with outcome in traumatic brain injury patients. Acta Neurochir..

[97] Uryga A., Ziółkowski A., Kazimierska A., Pudełko A., Mataczyński C., Lang E.W., Czosnyka M., Kasprowicz M. (2023). Analysis of intracranial pressure pulse waveform in traumatic brain injury patients: A CENTER-TBI study. J. Neurosurg..

[98] Ghaith H.S., Nawar A.A., Gabra M.D., Abdelrahman M.E., Nafady M.H., Bahbah E.I., Ebada M.A., Ashraf G.M., Negida A., Barreto G.E. (2022). A Literature Review of Traumatic Brain Injury Biomarkers. Mol. Neurobiol..

[99] Nielson J.L., Cooper S.R., Yue J.K., Sorani M.D., Inoue T., Yuh E.L., Mukherjee P., Petrossian T.C., Paquette J., Lum P.Y. (2017). Uncovering precision phenotype-biomarker associations in traumatic brain injury using topological data analysis. PLoS ONE.

[100] Abdelhak A., Foschi M., Abu-Rumeileh S., Yue J.K., D’Anna L., Huss A., Oeckl P., Ludolph A.C., Kuhle J., Petzold A. (2022). Blood GFAP as an emerging biomarker in brain and spinal cord disorders. Nat. Rev. Neurol..

[101] Frankel M., Fan L., Yeatts S.D., Jeromin A., Vos P.E., Wagner A.K., Wolf B.J., Pauls Q., Lunney M., Merck L.H. (2019). Association of Very Early Serum Levels of S100B, Glial Fibrillary Acidic Protein, Ubiquitin C-Terminal Hydrolase-L1, and Spectrin Breakdown Product with Outcome in ProTECT III. J. Neurotrauma.

[102] Bazarian J.J., Biberthaler P., Welch R.D., Lewis L.M., Barzo P., Bogner-Flatz V., Gunnar Brolinson P., Büki A., Chen J.Y., Christenson R.H. (2018). Serum GFAP and UCH-L1 for prediction of absence of intracranial injuries on head CT (ALERT-TBI): A multicentre observational study. Lancet Neurol..

[103] Korley F.K., Jain S., Sun X., Puccio A.M., Yue J.K., Gardner R.C., Wang K.K.W., Okonkwo D.O., Yuh E.L., Mukherjee P. (2022). Prognostic value of day-of-injury plasma GFAP and UCH-L1 concentrations for predicting functional recovery after traumatic brain injury in patients from the US TRACK-TBI cohort: An observational cohort study. Lancet Neurol..

[104] Wang K.K., Yang Z., Zhu T., Shi Y., Rubenstein R., Tyndall J.A., Manley G.T. (2018). An update on diagnostic and prognostic biomarkers for traumatic brain injury. Expert. Rev. Mol. Diagn..

[105] Volovici V., Ercole A., Citerio G., Stocchetti N., Haitsma I.K., Huijben J.A., Dirven C.M.F., van der Jagt M., Steyerberg E.W., Nelson D. (2019). Variation in Guideline Implementation and Adherence Regarding Severe Traumatic Brain Injury Treatment: A CENTER-TBI Survey Study in Europe. World Neurosurg..

[106] Cnossen M.C., Scholten A.C., Lingsma H.F., Synnot A., Tavender E., Gantner D., Lecky F., Steyerberg E.W., Polinder S. (2021). Adherence to Guidelines in Adult Patients with Traumatic Brain Injury: A Living Systematic Review. J. Neurotrauma.

[107] English S.W., Turgeon A.F., Owen E., Doucette S., Pagliarello G., McIntyre L. (2013). Protocol management of severe traumatic brain injury in intensive care units: A systematic review. Neurocrit. Care.

[108] Dheansa S., Rajwani K.M., Pang G., Bench S., Kailaya-Vasan A., Maratos E., Lavrador J.P., Bhangoo R., Tolias C.M. (2023). Relationship between guideline adherence and outcomes in severe traumatic brain injury. Ann. R. Coll. Surg. Engl..

[109] Svedung Wettervik T.M., Lewén A., Enblad P. (2021). Fine Tuning of Traumatic Brain Injury Management in Neurointensive Care—Indicative Observations and Future Perspectives. Front. Neurol..

[110] Kompanje E.J., Maas A.I., Hilhorst M.T., Slieker F.J., Teasdale G.M. (2005). Ethical considerations on consent procedures for emergency research in severe and moderate traumatic brain injury. Acta Neurochir..

[111] Bekar A., Doğan S., Abaş F., Caner B., Korfali G., Kocaeli H., Yilmazlar S., Korfali E. (2009). Risk factors and complications of intracranial pressure monitoring with a fiberoptic device. J. Clin. Neurosci..

[112] Sahuquillo J., Biestro A. (2014). Is intracranial pressure monitoring still required in the management of severe traumatic brain injury? Ethical and methodological considerations on conducting clinical research in poor and low-income countries. Surg. Neurol. Int..

[113] Chesnut R.M., Temkin N., Carney N., Dikmen S., Rondina C., Videtta W., Petroni G., Lujan S., Pridgeon J., Barber J. (2012). A trial of intracranial-pressure monitoring in traumatic brain injury. N. Engl. J. Med..

[114] Stiver S.I. (2009). Complications of decompressive craniectomy for traumatic brain injury. Neurosurg. Focus.

[115] Coronado V.G., McGuire L.C., Sarmiento K., Bell J., Lionbarger M.R., Jones C.D., Geller A.I., Khoury N., Xu L. (2012). Trends in traumatic brain injury in the US and the public health response: 1995–2009. J. Saf. Res..

[116] Gilchrist J., Thomas K., Wald M., Langlois J. (2007). Nonfatal traumatic brain injuries from sports and recreation activities--United States, 2001–2005. MMWR Morb. Mortal. Wkly. Rep..

[117] Parker E.M., Gilchrist J., Schuster D., Lee R., Sarmiento K. (2015). Reach and knowledge change among coaches and other participants of the online course:“Concussion in sports: What you need to know”. J. Head. Trauma. Rehabil..

[118] Baldwin G., Breiding M., Sleet D. (2016). Using the public health model to address unintentional injuries and TBI: A perspective from the Centers for Disease Control and Prevention (CDC). NeuroRehabilitation.

[119] Lee R. (2017). The CDC’s STEADI initiative: Promoting older adult health and independence through fall prevention. Am. Fam. Physician.

[120] Casey C.M., Parker E.M., Winkler G., Liu X., Lambert G.H., Eckstrom E. (2017). Lessons learned from implementing CDC’s STEADI falls prevention algorithm in primary care. Gerontologist.

[121] Olson Z., Staples J.A., Mock C., Nguyen N.P., Bachani A.M., Nugent R., Verguet S. (2016). Helmet regulation in Vietnam: Impact on health, equity and medical impoverishment. Inj. Prev..

[122] Popescu C., Anghelescu A., Daia C., Onose G. (2015). Actual data on epidemiological evolution and prevention endeavours regarding traumatic brain injury. J. Med. Life.

[123] Fatuki T.A., Zvonarev V., Rodas A.W., Bellman V., Rodas A. (2020). Prevention of traumatic brain injury in the United States: Significance, new findings, and practical applications. Cureus.

[124] Fong N., Feng J., Hubbard A., Dang L.E., Pirracchio R. (2024). IntraCranial pressure prediction AlgoRithm using machinE learning (I-CARE): Training and Validation Study. Crit. Care Explor..

[125] Myers R.B., Lazaridis C., Jermaine C.M., Robertson C.S., Rusin C.G. (2016). Predicting Intracranial Pressure and Brain Tissue Oxygen Crises in Patients With Severe Traumatic Brain Injury. Crit. Care Med..

[126] Hu X., Xu P., Asgari S., Vespa P., Bergsneider M. (2010). Forecasting ICP elevation based on prescient changes of intracranial pressure waveform morphology. IEEE Trans. Biomed. Eng..

[127] Schroder A., Lawrence T., Voets N., Garcia-Gonzalez D., Jones M., Peña J.M., Jerusalem A. (2021). A Machine Learning Enhanced Mechanistic Simulation Framework for Functional Deficit Prediction in TBI. Front. Bioeng. Biotechnol..

[128] Raj R., Luostarinen T., Pursiainen E., Posti J.P., Takala R.S.K., Bendel S., Konttila T., Korja M. (2019). Machine learning-based dynamic mortality prediction after traumatic brain injury. Sci. Rep..

[129] Raj R., Wennervirta J.M., Tjerkaski J., Luoto T.M., Posti J.P., Nelson D.W., Takala R., Bendel S., Thelin E.P., Luostarinen T. (2022). Dynamic prediction of mortality after traumatic brain injury using a machine learning algorithm. npj Digit. Med..

[130] Courville E., Kazim S.F., Vellek J., Tarawneh O., Stack J., Roster K., Roy J., Schmidt M., Bowers C. (2023). Machine learning algorithms for predicting outcomes of traumatic brain injury: A systematic review and meta-analysis. Surg. Neurol. Int..

[131] Mainali S., Park S. (2023). Artificial Intelligence and Big Data Science in Neurocritical Care. Crit. Care Clin..

[132] GBD 2016 Traumatic Brain Injury and Spinal Cord Injury Collaborators (2019). Global, regional, and national burden of traumatic brain injury and spinal cord injury, 1990–2016: A systematic analysis for the Global Burden of Disease Study 2016. Lancet Neurol.

[133] Yue J.K., Winkler E.A., Sharma S., Vassar M.J., Ratcliff J.J., Korley F.K., Seabury S.A., Ferguson A.R., Lingsma H.F., Deng H. (2017). Temporal profile of care following mild traumatic brain injury: Predictors of hospital admission, follow-up referral and six-month outcome. Brain Inj..

[134] Maas A.I.R., Menon D.K., Adelson P.D., Andelic N., Bell M.J., Belli A., Bragge P., Brazinova A., Büki A., Chesnut R.M. (2017). Traumatic brain injury: Integrated approaches to improve prevention, clinical care, and research. Lancet Neurol..

[135] Jiang J.Y., Gao G.Y., Feng J.F., Mao Q., Chen L.G., Yang X.F., Liu J.F., Wang Y.H., Qiu B.H., Huang X.J. (2019). Traumatic brain injury in China. Lancet Neurol..

[136] Amorim R.L., Oliveira L.M., Malbouisson L.M., Nagumo M.M., Simoes M., Miranda L., Bor-Seng-Shu E., Beer-Furlan A., De Andrade A.F., Rubiano A.M. (2019). Prediction of Early TBI Mortality Using a Machine Learning Approach in a LMIC Population. Front. Neurol..

[137] Clavijo A., Khan A.A., Mendoza J., Montenegro J.H., Johnson E.D., Adeleye A.O., Rubiano A.M. (2019). The Role of Decompressive Craniectomy in Limited Resource Environments. Front. Neurol..

[138] Kanmounye U.S. (2021). The Rise of Inflow Cisternostomy in Resource-Limited Settings: Rationale, Limitations, and Future Challenges. Emerg. Med. Int..

